# Ultrasounic-radiomics models for predicting the response to Atezolizumab plus Bevacizumab in patients with unresectable hepatocellular carcinoma

**DOI:** 10.1371/journal.pone.0334099

**Published:** 2025-10-29

**Authors:** Yiran Li, Zonghan Liu, Yi Qian, Kang Wang, Yijun Gu, Yan Chen, Haozheng Jiang, Shuqun Cheng, Dong Jiang

**Affiliations:** 1 Department of Ultrasonography, Shanghai Eastern Hepatobiliary Surgery Hospital, Shanghai, China; 2 Department of Hepatic Surgery VI, Shanghai Eastern Hepatobiliary Surgery Hospital, Shanghai, China; 3 Medical Anthropology, Psychology, College of Art and Science Department, Case Western Reserve University, Cleveland, OH, USA; Sun Yat-Sen University, CHINA

## Abstract

**Background:**

Atezolizumab plus Bevacizumab is an effective treatment for unresectable hepatocellular carcinoma, but the assessment methods are limited.

**Objective:**

To establish an early predictive model using Ultrasounic-radiomics (UR) for predicting the therapeutic efficacy of Atezolizumab plus Bevacizumab in unresectable hepatocellular carcinoma.

**Methods:**

We retrospectively analyzed 170 patients with unresectable hepatocellular carcinoma, extracting 1560 imaging features pre- and one-week post-treatment. Separate UR models were established to predict treatment efficacy. Model performance was evaluated using calibration curves and the area under the receiver operating characteristic curve (AUC).

**Results:**

Two UR models were ultimately established. The pre-treatment UR model achieved an AUC of 0.790 in the train group and 0.706 in the validation group. The post-treatment UR model achieved an AUC of 0.855 in the train group and 0.704 in the validation group. Using a cutoff value of 0.528 to divide patients into high-risk and low-risk groups, the Kaplan-Meier survival curves showed statistically significant differences between the two groups. The hazardous and moderate-risk groups’ Kaplan-Meier survival curves revealed statistically significant distinctions.

**Conclusion:**

The UR models show promise in predicting the efficacy and prognosis of combined targeted therapy and immunotherapy in unresectable hepatocellular carcinoma, particularly highlighting the importance of ultrasound assessments three months post-treatment.

## Introduction

Hepatocellular carcinoma ranks as the sixth most frequently diagnosed carcinoma globally and is a major contributor to mortality [1]. Surgical resection is still the first-line treatment for hepatocellular carcinoma, but most cases are at an advanced stage when first diagnosed. Treatment of unresectable hepatocellular carcinoma is a complex process aimed at prolonging patient survival, alleviating symptoms, and improving quality of life [2]. Common treatment regimens for unresectable hepatocellular carcinoma include chemotherapy, radiotherapy, transarterial chemoembolization (TACE), targeted therapy, and immunotherapy [3]. In recent years, significant efficacy has been achieved with targeted therapy and immunotherapy drugs for unresectable hepatocellular carcinoma [4]. Clinical trials and real-world applications have observed marked responses and extended survival periods in some patients treated with these drugs.

With the disclosure of IMbrave 150, both atezolizumab and bevacizumab in conjunction with one another has been the first-line treatment for unresectable hepatocellular carcinoma [5]. According to current research and clinical trial data, the overall response rate of atezolizumab plus bevacizumab in unresectable hepatocellular carcinoma patients may range from 20% to 40% [6]. However, factors such as the patient’s immune status, tumor burden, and tumor genomic variations also influence the efficacy [7]. Some patients may have a more active immune system and respond better, while others may exhibit immune escape mechanisms or drug resistance, leading to poorer treatment outcomes [8].

Currently, the methods for assessing the efficacy of atezolizumab plus bevacizumab in unresectable hepatocellular carcinoma involve using imaging techniques such as CT scans and MRI to examine the size, number, and distribution of tumors, which can evaluate the direct impact of targeted therapy and immunotherapy on the tumor [9]. When treating unresectable hepatocellular carcinoma, the overall survival, or OS, and PFS, or progression-free survival, of those being treated can be used to evaluate the performance of atezolizumab together with bevacizumab [10]. Measuring immune indicators in patients, such as T-cell activity, tumor-associated antigen levels, cytokine levels, etc., can evaluate the impact on the immune system and treatment efficacy [11,12]. However, methods that predict the efficacy of atezolizumab plus bevacizumab in unresectable hepatocellular carcinoma are still lacking.

Radiomics is a method that utilizes medical imaging data for systematic quantitative analysis and pattern recognition. In assessing the efficacy of unresectable hepatocellular carcinoma treatment, radiomics can utilize ultrasound, CT scans, or MRI for tumor evaluation [13,14]. Radiomics not only allows for the quantitative measurement of tumor size and volume but also captures objective image features to monitor changes in the tumor before and after treatment. It can capture vascular characteristics of the tumor, such as blood perfusion and angiogenesis, reflecting changes in the vasculature after treatment [15].

Given the widespread application of radiomics in tumor staging, early diagnosis, disease differentiation, prognosis prediction, and treatment evaluation, its clinical value has been demonstrated in various malignancies. For example, CT-based radiomics models have achieved high accuracy in differentiating early- from late-stage pancreatic ductal adenocarcinoma, providing a promising noninvasive tool for improving early diagnosis and prognostic assessment [16]. Similarly, CT radiomics has been shown to effectively characterize tumor heterogeneity and aid in predicting histologic grade and guiding treatment decisions in pancreatic cancer [17]. These findings further support the potential clinical utility of radiomics in hepatocellular carcinoma management.

Furthermore, recent comparative studies have highlighted that multi-modality radiomics, such as combining DCE-MRI, B-mode ultrasound, and strain elastography, can achieve superior diagnostic performance compared with single-modality models, particularly in differentiating benign from malignant lesions (AUCs ≈ 0.95), outperforming traditional BI-RADS assessment methods [18]. Similarly, CT- and MRI-based radiomics have shown promising accuracy in tumor characterization, treatment response prediction, and prognostic assessment in various cancers, including ovarian and liver malignancies. However, methodological standardization remains an ongoing challenge [19,20].

In contrast, ultrasound-based radiomics offers distinct advantages, including being radiation-free, widely accessible, and cost-effective. However, its reproducibility can be affected by variability in acquisition parameters, operator dependency, and image pre-processing, which must be carefully addressed to ensure reliable feature extraction [21,22]. These comparative insights provide a valuable context for positioning ultrasound radiomics within the broader spectrum of imaging-based predictive modeling.

Therefore, this study aims to establish a pre- and post-treatment ultrasound radiomics prediction model to predict the efficacy of atezolizumab plus bevacizumab in unresectable hepatocellular carcinoma. This will provide evidence and evaluation indicators in clinical practice.

## Materials and methods

### Population

**Fig 1:**
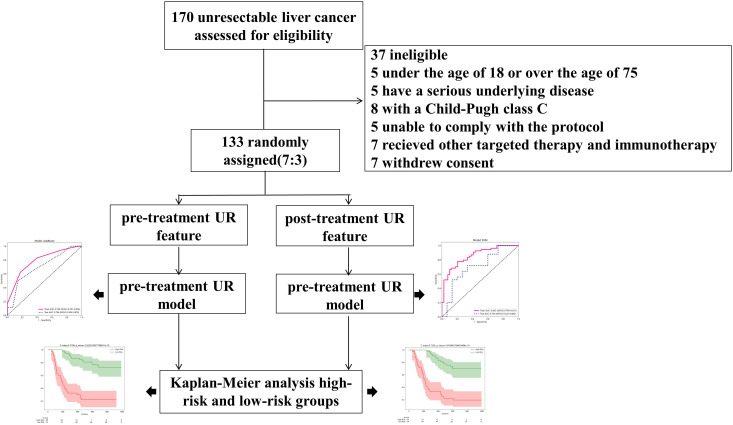
The Flow chart is an overview of the screening process(UR = Ultrasounic-radiomics).

Within the timeframe spanning May 2021 to December 2023, the Eastern Hepatobiliary Surgery Hospital admitted 170 patients with clinically and histologically diagnosed hepatocellular carcinoma, all of whom were not amenable to surgical or locoregional therapy [23]. Individuals were enrolled if they met the following criteria: age between 18 and 75 years; Child-Pugh Class A or B; Had an Eastern Cooperative Oncology Group (ECOG) performance status of 0 or 1; at least a single lesion that meets the mRECIST standards for measurement, without/with metastasis; no prior immunotherapy or targeted therapy. Individuals were excluded from enrollment if they had received other anti-tumor therapy during the procedure; had serious organ dysfunctions like cardiopulmonary insufficiency; had active infection, autoimmune disease or other disease requiring long-term hormones or immunosuppressive treatment; had serious mental illness. This meticulous selection process culminated in the inclusion of 93 patients in the study, facilitating the formulation of a predictive model, and subsequent internal validation was executed utilizing a subset of 40 patients (Fig 1). The Ethics Committee of the aforementioned hospital bestowed approval upon this retrospective study, corroborated by written informed consent acquired from all patient participants (Ethics Number: EHBHKY2023-K034-P001). The research was conducted in accordance with the Declaration of Helsinki.

### Targeted therapy and immunotherapy

The specific implementation process usually includes the following steps: Every three weeks, enrolled patients get intravenous injections of a dose of 1,200 mg of atezolizumab along with 15 mg/kg of bevacizumab; Evaluation is conducted after every 3 months [24]. Once patients achieve successful conversion and downstaging, surgical treatment and postoperative consolidation therapy are performed with the informed consent of the patient and their family. If no response and disease progression or loss of clinical benefit as determined by investigators, switching to a second-line treatment regimen is recommended [25].

### Follow-up and clinical observation indicators

Every patient underwent consistent monitoring in the outpatient setting. Routine procedures entailed conducting physical evaluations and laboratory assays, of which alpha-fetoprotein (AFP) quantification and ultrasound evaluations stood out, occurring with a frequency spanning 3–6 months. Imaging studies, such as CT and MRI, were customarily scheduled semiannually. When there was evidence suggesting tumor recurrence, treatment modalities like TACE or ablation were contemplated and chosen based on the specific attributes of the tumor. The statistical inferences were grounded on datasets accrued prior to December 1, 2023.

Imaging changes: including changes in the size of intrahepatic and extrahepatic tumors, tumor and vascular thrombus necrosis; changes in quantitative values of alpha-fetoprotein (AFP); other indicators: disease control rate (DCR), transition to surgical treatment rate, and objective response rate (ORR), pathological complete response rate (pCR), as well as adverse reactions. Adverse reactions to be observed include hypertension, hand-foot syndrome, diarrhea, rash, cutaneous capillary proliferation, exfoliative dermatitis, electrolyte imbalance and significant organ toxicities.

### Ultrasound examination

Utilizing the Acuson Sequoia diagnostic ultrasound apparatus (Siemens Healthineers, Mountain View, California, USA) paired with a 5C1 convex abdominal transducer, conventional ultrasound diagnostics were performed. All ultrasound examinations were conducted with standardized machine parameters to ensure consistency across sessions and operators: the dynamic range was fixed at 60 dB, the transducer frequency was set to 3.5–5.0 MHz (center frequency 4.0 MHz), and the gain was adjusted to a baseline of 50% with a permissible variation of ±5% based on patient body habitus (recorded in detail for each examination). Prior to the procedure, patients underwent a fasting period spanning a minimum of 8 hours. Liver ultrasonography targeting solid liver anomalies was conducted by a seasoned sonographer boasting over a decade and a half in expertise. Subsequent to this standard ultrasound protocol, another specialist adept in ultrasound evaluations using the aforementioned apparatus and transducer rechecked the parameter settings to confirm adherence to the standardized protocol, with any deviations documented and justified. The patient is required to undergo ultrasound examination prior to treatment, and outpatient or inpatient ultrasound examination should be conducted 3–4 cycles after treatment.

### Radiomic features extraction

Three categories can be used to group the created features: (I) geometry, (II) intensity, and (III) texture. The tumor’s shape in three dimensions characteristics are described by its geometry features. The initial-order mathematical distribution of the region intensities inside the tumor is defined by the intensity characteristics. The designs, or the following- and high-order spatial distributions of the intensities, are outlined by the texture traits. Here, a variety of techniques are used to extract the texture traits, such as the neighborhood gray-tone difference matrix (NGTDM), gray-level co-occurrence matrix (GLCM), gray-level run length matrix (GLRLM), and gray-level size zone matrix (GLSZM). Radiomic features were extracted using the open-source PyRadiomics toolkit (version 3.0.1, https://pyradiomics.readthedocs.io) [26], with parameters optimized for grayscale ultrasound images. The in-house feature assessment algorithm was developed in Python (v3.8) and comprised several modules, including image normalization (z-score), stability testing, redundancy removal using Spearman correlation (threshold > 0.9), and LASSO-based selection via scikit-learn (v0.24.2). The final radiomics signatures were constructed using a linear combination of the retained features weighted by their LASSO coefficients.

### Feature selection

Our analysis takes three steps: (1) We performed feature scanning on every radiomic characteristics and the Mann-Whitney Utest statistical analysis. Only the radiomic characteristic with a pvalue<0.05 was retained. (2) One of the features with a measurement of correlation more than 0.9 between both of the characteristics is kept. The Spearman’s rank correlation coefficient was used as well to figure out the association between features for features that had excellent repeatability. We employ a greedy recursive deletion technique for feature screening, meaning that the feature with the most redundancy in the present set is removed each time, in order to preserve the capacity to display aspects to the maximum extent possible. A few aspects were eventually retained afterwards this. (3) On the discovery data set, the least absolute shrinkage and selection operator (LASSO) regression model was applied in order to develop signatures. LASSO sets the coefficients for several unimportant features exactly to zero and decreases all regression coefficients towards zero, subject to the regulation weight λ. A 10-fold cross validation using a minimum criterion was used to determine the ideal λ, with the final outcome of λ producing the lowest cross validation error. Regression model fitting was performed using the remaining features that had positive coefficients, and the resulting radiomics signature was created. We next used a linear mixture of preserved features evaluated by their model coefficients to get a radiomics score for each patient. The LASSO-based regression modeling program for Python was utilized, called scikit-learn [27].

### Machine learning

We fed the ultimate features into the machine training for risk model generation after Lasso characteristic screening. Here, we adopt 5 fold cross validation to obtain the final Rad Signature. The final selection and establishment of the most suitable machine learning model for radiomic data classification was performed.

### Statistical methods

For the data analytics, both IBM’s SPSS Statistics 28 and the R software suite (version 4.0.3, sourced from the R Foundation for Statistical Computing in Vienna, Austria) were utilized. Parameters of a continuous nature got denoted as mean ± standard deviations. Such parameters underwent comparative evaluations using the t test or alternatively, the Mann-Whitney U test. Variables were segregated on clinical outcome bases, with their summaries including both numerical counts and relative proportions. Fisher’s exact test or the chi-square test became the statistical tools of choice for their analysis. A small proportion of missing data (<5% of the total dataset) was observed, primarily in laboratory indicators and follow-up imaging details. Missing values were imputed using multiple imputation with 10 iterations via the “mice” package in R to maintain statistical power and reduce bias. Three cases with critical missing data (e.g., absence of both pre- and post-treatment ultrasound data, essential for radiomic feature extraction and model validation) were excluded from the final analysis. Employing an SVM approach, all influential factors were taken into consideration. Nonetheless, the multivariable SCM model integrated solely those factors which exhibited statistical relevance (*P* < 0.05). This integration relied on a progressive sequential methodology encompassing odds ratios (ORs) computations. Only those variables demonstrating an area under the curve (AUC) exceeding 0.7 held significance. The constructed nomogram’s efficacy underwent assessment through the concordance index (C-index). R software facilitated the validation of this model using a separate validation subset.

## Results

### Demographics of patients

We examined the demographic details of patients in the train and test groups, as shown in Table 1. Multiple characteristics, such as age, height, and weight, were compared between the patient groups, and no statistically significant differences were observed (*P* > 0.05). Subsequent analysis incorporated imaging indicators that exhibited significant differences.

**Table 1:** Baseline characteristics and treatment of adverse reactions of patients in two cohorts.

Characters	Train cohort				Test cohort			
	ALL (n = 93)	Effective (n = 65)	Without effective (n = 18)	*P* value	ALL (n = 93)	Effective (n = 65)	Without effective (n = 18)	*p* value
**General Data**								
Age (y)	50.16 ± 10.81	51.41 ± 9.72	48.44 ± 12.08	0.193	49.83 ± 9.34	48.40 ± 8.83	52.20 ± 1.00	0.217
Heigth (cm)	165.06 ± 7.71	166.44 ± 7.73	163.15 ± 7.35	0.051	164.95 ± 5.81	166.28 ± 5.65	162.73 ± 5.56	0.061
Weight (kg)	65.35 ± 9.56	66.21 ± 9.51	64.15 ± 9.63	0.308	65.25 ± 9.48	66.90 ± 9.80	63.83 ± 7.21	0.052
Sex				0.057				0.170
Man	52(55.9%)	35(64.8%)	17(43.6%)		29(72.5%)	20(80%)	9(60%)	
Female	41(44.1%)	19(35.2%)	22(56.4%)		11(27.5%)	5(20%)	6(40%)	
Drinking				0.872				0.261
Yes	9(9.7%)	5(9.3%)	4(10.3%)		2(5%)	2(8%)	0(0%)	
No	84(90.3%)	49(90.7%)	35(89.7%)		38(95%)	23(92%)	15(100%)	
Smoking				0.418				0.064
Yes	10(10.8%)	7(13%)	3(7.7%)		5(12.5%)	5(20%)	0(0%)	
No	83(89.2%)	47(87%)	36(92.3%)		35(87.5%)	20(80%)	15(100%)	
DM				0.584				0.586
Yes	10(10.8%)	5(9.3%)	5(12.8%)		4(10%)	3(12%)	1(6.7%)	
No	83(89.2%)	49(90.7%)	34(87.2%)		36(90%)	22(88%)	14(93.3%)	
hypertension				0.740				0.902
Yes	13(14%)	7(13%)	6(15.4%)		5(12.5%)	3(12%)	2(13.3%)	
No	80(86%)	47(87%)	33(84.6%)		35(87.5%)	22(88%)	13(867%)	
Hepatitis				0.384				0.449
HBV	75(80.6%)	45(83.3%)	30(76.9%)		32(80%)	20(80%)	12(80%)	
HCV	4(4.3%)	3(5.6%)	1(2.6%)		2(5%)	2(8%)	0(0%)	
Other	14(15.1%)	6(11.1%)	8(20.5%)		6(15%)	3(12%)	3(20%)	
Hepatocirrhosis				0.923				0.505
Yes	72(77.4%)	42(58.3%)	30(76.9%)		24(60%)	14(56%)	10(66.7%)	
No	21(22.6%)	12(22.2%)	9(23.1%)		16(40%)	11(44%)	5(33.3%)	
Extrahepatic metastasis				0.524				0.505
Yes	37(39.8%)	20(37%)	17(43.6%)		16(40%)	9(36%)	7(46.7%)	
No	56(60.2%)	34(63%)	22(56.4%)		24(60%)	16(64%)	8(53.3%)	
Child-Pugh grade				0.445				0.462
A	52(55.9%)	32(59.3%)	20(51.3%)		19(47.5%)	13(52%)	6(40%)	
B	41(44.1%)	22(40.7%)	19(48.7%)		21(52.5%)	12(48%)	9(60%)	
Pre-AFP	361.41 ± 530.76	414.07 ± 545.46	282.42 ± 503.44	0.251	436.97 ± 564.88	490.76 ± 581.70	350.92 ± 545.26	0.459
Post-AFP	182.14 ± 399.80	81.53 ± 271.37	333.04 ± 507.70	0.012	207.46 ± 433.03	62.38 ± 234.48	383.63 ± 551.46	0.037
Adverse reaction								
Hypertension				0.940				0.747
Yes	14(15.1%)	8(14.8%)	6(15.4%)		6(15%)	4(16%)	3(20%)	
No	79(84.9%)	46(85.2%)	33(84.6%)		34(85%)	21(84%)	12(80%)	
Hand-foot syndrome				0.959				0.163
Yes	7(7.5%)	4(7.4%)	3(7.7%)		3(7.5%)	3(12%)	0(0%)	
No	86(92.5%)	5(92.6%)	36(92.3%)		37(92.5%)	22(88%)	15(100%)	
Diarrhea				0.959				0.877
Yes	7(7.5%)	4(7.4%)	3(7.7%)		3(7.5%)	2(8%)	1(6.7%)	
No	86(92.5%)	5(92.6%)	36(92.3%)		37(92.5%)	23(92%)	14(93.3%)	
Skin rash				0.928				0.261
Yes	5(5.4%)	3(5.6%)	2(5.1%)		2(5%)	2(8%)	0(0%)	
No	88(94.6%)	51(94.4%)	37(94.9%)		38(95%)	23(92%)	15(100%)	
Exfoliative dermatitis				0.400				0.708
Yes	5(5.4%)	2(3.7%)	3(7.7%)		2(5%)	1(4%)	1(6.7%)	
No	88(94.6%)	52(96.3%)	36(92.3%)		38(95%)	24(96%)	14(93.3%)	
Cutaneous capillary hyperplasia				0.928				0.708
Yes	5(5.4%)	2(3.7%)	3(7.7%)		2(5%)	1(4%)	1(6.7%)	
No	88(94.6%)	52(96.3%)	36(92.3%)		38(95%)	24(96%)	14(93.3%)	
Electrolyte turbulence Disorder				0.307				0.261
Yes	5(5.4%)	4(7.4%)	1(2.6%)		2(5%)	2(8%)	0(0%)	
No	88(94.6%)	50(92.6%)	38(97.4%)		38(95%)	23(92%)	15(100%)	

Note—Pre-AFP = pre-treatment alpha-fetoprotein, Post-AFP = post-treatment alpha-fetoprotein, P value for comparisons between effective and without effective patients.

### The results and adverse effects

According to the mRECIST criteria, the evaluation of treatment response for conversion therapy categorizes complete response (CR), partial response (PR), and stable disease (SD) as effective, while disease progression (PD) is considered as without effective. In the train set, 54 cases (58%) were classified as effective, and 39 cases (42%) as without effective. In the test set, 25 cases (62.5%) were classified as effective, and 15 cases (37.5%) as without effective. Common adverse reactions are detailed in Table 1, and no significant major organ toxicities were observed in both groups. Symptomatic treatment was effective in managing adverse reactions, and no reduction in drug dosage was required, except for 3 cases of diarrhea and 5 cases of desquamation dermatitis where reducing the targeted drug dosage was necessary. As of the specified data cutoff date, 54 cases experienced disease recurrence, and 64 cases resulted in death. As of November 2023, the longest observed survival after treatment was 996 days, and the overall survival (OS) and recurrence-free survival (RFS) endpoints have not yet been reached for patients.

### Extraction of ultrasound imaging-based radiomics features

**Fig 2:**
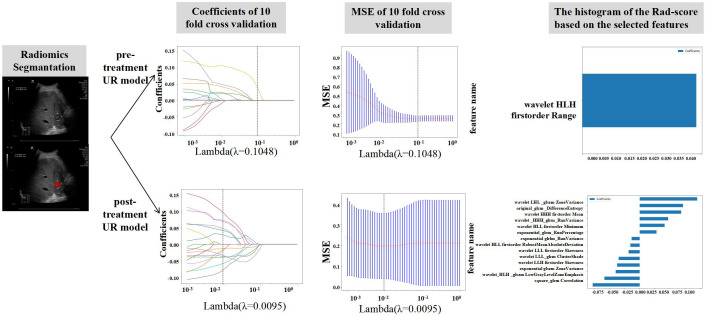
Extraction of pre-treatment and post-treatment ultrasound radiomics features.

A total of 1560 handmade characteristics are acquired: 14 form characteristics, 306 firstorder characteristics, and texture characteristics round out the total. An in-house feature assessment algorithm developed in Pyradiomics (http://pyradiomics.readthedocs.io) is used to extract all custom features. The Rad-score with a least absolute contraction and choosing operator (LASSO) logistic regression framework was developed by selecting nonzero parameters. Fig 2 displays the coefficients and MSE (mean standard error) of the 10-fold verification. The coefficients value in the final none-zero features that were chosen is displayed. The Rad-score histogram according to the chosen characteristics. Both prior to and following therapy, the chosen characteristics and correlations were checked (Table 2).

**Table 2:** Selected features and coefficients after screening.

Sequence	Name
pre-treatment Ultrasounic-radiomics model	‘wavelet_HLH_firstorder_Range’
post-treatment Ultrasounic-radiomics model	‘exponential_glrlm_RunPercentage’, ‘exponential_glrlm_RunVariance’, ‘exponential_glszm_ZoneVariance’, ‘original_glcm_DifferenceEntropy’, ‘square_glcm_Correlation’, ‘wavelet_HHH_firstorder_Mean’, ‘wavelet_HHH_glrlm_RunVariance’, ‘wavelet_HLH_glszm_LowGrayLevelZoneEmphasis’, ‘wavelet_HLL_firstorder_Minimum’, ‘wavelet_HLL_firstorder_RobustMeanAbsoluteDeviation’, ‘wavelet_LHL_glszm_ZoneVariance’, ‘wavelet_LLH_firstorder_Skewness’, ‘wavelet_LLL_firstorder_Skewness’, ‘wavelet_LLL_glcm_ClusterShade’

### Modeling of radiomics features

**Fig 3:**
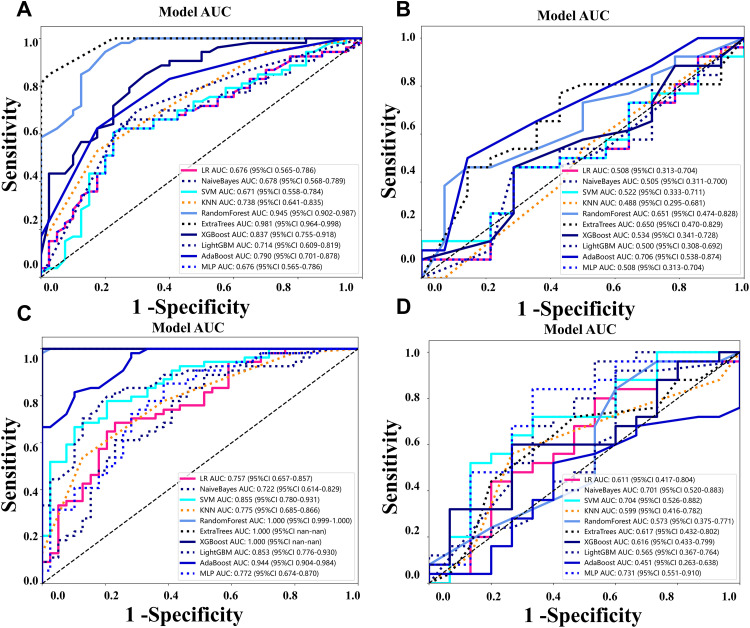
The area under the curve (AUC) of the radiomics signature model on both the train (A and B) and test cohorts (C and D).

The optimal model was established based on the characteristics of radiomics, and compared with other classifiers such as Logistic regression (LR), support vector machine (SVM) and random forest (RF). Selecting the most appropriate model for predicting the efficacy of targeted surface treatment in unresectable hepatocellular carcinoma, the pre-treatment UR model is shown in Table 3, while the post-treatment UR model is presented in Table 4. Adaboost was selected for establishing the predictive model prior to treatment, while SVM was chosen for constructing the predictive model post-treatment. Adaboost was selected for the pre-treatment model and SVM for the post-treatment model. Although ExtraTrees and RandomForest showed favorable NPV and accuracy, AdaBoost and SVM were chosen for their greater cross-validation stability, clinical relevance, and compatibility with selected radiomic features. In particular, AdaBoost offered higher PPV, important for minimizing false positives before treatment, while SVM provided a balanced performance and reduced overfitting risk post-treatment. Fig 3 illustrates the area under the curve (AUC) of the radiomics signature model on both the train and test cohorts.

**Table 3:** Evaluation of pre-treatment models using different machine learning algorithms.

Model name	Accuracy	AUC	95% CI	Sensitivity	Specificity	PPV	NPV	Precision
Train-LR	0.667	0.676	0.565-0.786	0.585	0.775	0.775	0.585	0.775
Test-LR	0.500	0.508	0.313−0.704	0.385	0.714	0.714	0.385	0.714
Train-NaiveBayes	0.677	0.678	0.568-0.789	0.623	0.750	0.767	0.600	0.767
NaiveBayes	0.500	0.505	0.311-0.700	0.385	0.714	0.714	0.385	0.714
Train-SVM	0.667	0.671	0.558-0.784	0.585	0.775	0.775	0.585	0.775
Test-SVM	0.500	0.522	0.333−0.711	0.385	0.714	0.714	0.385	0.714
Train-KNN	0.505	0.738	0.641-0.835	0.132	1.000	1.000	0.465	1.000
Test-KNN	0.450	0.488	0.295−0.681	0.385	0.571	0.625	0.333	0.625
Train-RandomForest	0.860	0.945	0.902-0.987	0.868	0.850	0.885	0.829	0.885
Test-RandomForest	0.575	0.651	0.474-0.828	0.385	0.929	0.909	0.448	0.909
Train-ExtraTrees	0.892	0.981	0.964−0.998	0.811	1.000	1.000	0.800	1.000
Test-ExtraTrees	0.625	0.650	0.470-0.829	0.654	0.571	0.739	0.471	0.739
Train-XGBoost	0.763	0.837	0.755-0.918	0.792	0.725	0.792	0.725	0.792
Test-XGBoost	0.525	0.534	0.341-0.728	0.423	0.714	0.733	0.400	0.733
Train-LightGBM	0.677	0.714	0.609-0.819	0.623	0.750	0.767	0.600	0.767
Test-LightGBM	0.350	0.500	0.308-0.692	0.115	0.786	0.500	0.324	0.500
**Train-AdaBoost**	**0.527**	**0.790**	**0.701-0.878**	**0.170**	**1.000**	**1.000**	**0.476**	**1.000**
**Test-AdaBoost**	**0.400**	**0.706**	**0.538-0.874**	**0.115**	**0.929**	**0.750**	**0.361**	**0.750**
Train-MLP	0.667	0.676	0.565-0.786	0.585	0.775	0.775	0.585	0.775
Test-MLP	0.500	0.508	0.313−0.704	0.385	0.714	0.714	0.385	0.714

Note- AUC = Area Under Curve, CI = confidence interval, PPV = positive predictive value, NPV = negative predictive value.

**Table 4:** Evaluation of post-treatment models using different machine learning algorithms.

Model name	Accuracy	AUC	95% CI	Sensitivity	Specificity	PPV	NPV	Precision
Train-LR	0.710	0.757	0.6570 - 0.8567	0.667	0.769	0.800	0.625	0.800
Test-LR	0.650	0.611	0.4169 - 0.8044	0.760	0.467	0.704	0.538	0.704
Train-NaiveBayes	0.667	0.722	0.6142 - 0.8293	0.593	0.769	0.780	0.577	0.780
Test-NaiveBayes	0.750	0.701	0.5196 - 0.8831	0.920	0.467	0.742	0.778	0.742
Train-SVM	0.774	0.855	0.7796 - 0.9308	0.759	0.795	0.837	0.705	0.837
Test-SVM	0.625	0.704	0.5265 - 0.8815	0.480	0.867	0.857	0.500	0.857
Train-KNN	0.495	0.775	0.6852 - 0.8656	0.130	1.000	1.000	0.453	1.000
Test-KNN	0.400	0.599	0.4156 - 0.7818	0.120	0.867	0.600	0.371	0.600
Train-RandomForest	0.925	1.000	0.9991 - 1.0000	0.870	1.000	1.000	0.848	1.000
Test-RandomForest	0.600	0.573	0.3753 - 0.7714	0.680	0.467	0.680	0.467	0.680
Train-ExtraTrees	0.419	1.000	1.0000 - 1.0000	0.000	1.000	0.000	0.419	0.000
Test-ExtraTrees	0.625	0.617	0.4324 - 0.8023	0.640	0.600	0.727	0.500	0.727
Train-XGBoost	0.989	1.000	1.000−1.000	0.981	1.000	1.000	0.975	1.000
Test-XGBoost	0.625	0.616	0.433−0.799	0.560	0.733	0.778	0.500	0.778
Train-LightGBM	0.785	0.853	0.776−0.930	0.759	0.821	0.854	0.711	0.854
Test-LightGBM	0.700	0.565	0.367−0.764	0.880	0.400	0.710	0.667	0.710
Train-AdaBoost	0.839	0.944	0.904-0.984	0.796	0.897	0.915	0.761	0.915
Test-AdaBoost	0.525	0.451	0.263-0.638	0.480	0.600	0.667	0.409	0.667
Train-MLP	0.742	0.772	0.674−0.871	0.833	0.615	0.750	0.727	0.750
Test-MLP	0.750	0.731	0.551- 0.910	0.800	0.667	0.800	0.667	0.800

Note- AUC = Area Under Curve, CI = confidence interval, PPV = positive predictive value, NPV = negative predictive value.

### Evaluation of the radiomics models

**Fig 4:**
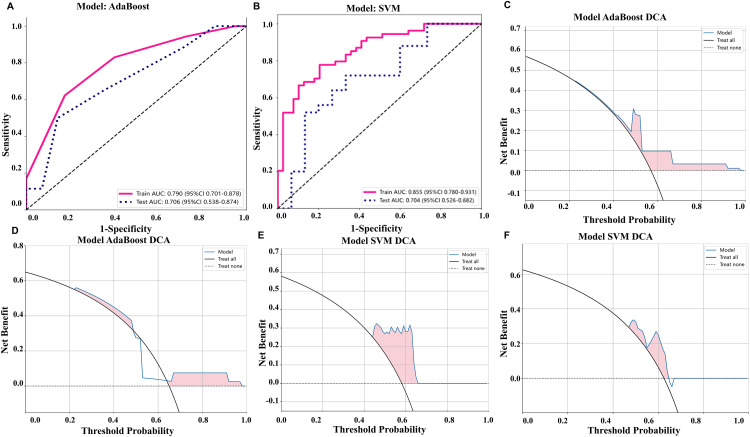
Evaluate the predictive performance of the model: ROC analysis of models on rad signature in pre-treatment US model (A) and post-treatment US model (B); Decision curve of in train and test cohort in pre-treatment US model (C and D) and post-treatment US (E and F).

**Fig 5:**
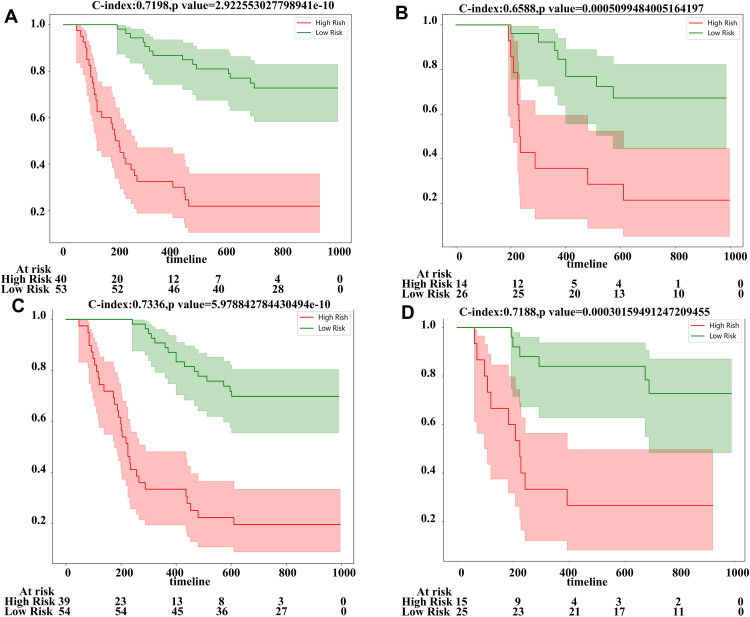
Pre-treatment US model and post-treatment US model predict patient survival: 10 KM (divided into high and low-risk groups based on rad-score cutoff values of 0.528 (train) and 0.528 (test)). The modeling group (A) and validation group (B) of the pre-treatment omics model can differentiate the two groups, while the modeling group (C) and validation group (D) of the post-treatment omics model can also differentiate the two groups (log rank less than 0.001).

In the pre-treatment model, the AUC of the modeling group was 0.790 (95%CI: 0.701–0.878), and in the test group, it was 0.705 (95%CI: 0.538–0.874). In the post-treatment model, the AUC of the modeling group was 0.855 (95%CI: 0.760–0.931), and in the test group, it was 0.704 (95%CI: 0.526–0.882), as shown in Fig 4A and 4B. Additionally, we performed decision curve analysis (DCA) to assess each model’s performance, which is demonstrated in Fig 4C–4F. Compared to scenarios where no prediction model was used (i.e., treat-all or treat-none scheme), the radiomic model exhibited a significant benefit for intervention in patients with a prediction probability. Using a cutoff value of 0.528 to divide patients into high-risk and low-risk groups, the Kaplan-Meier survival curves showed statistically significant differences between the two groups (log rank p-values were both less than 0.001) (Fig 5).

## Discussion

Although surgical resection remains the primary treatment for early-stage hepatocellular carcinoma, many patients require systemic therapy due to high recurrence rates and advanced diagnosis. Despite systemic therapy, longevity chances remain dismal. The body’s immune response is essential in inhibiting the advancement of cancer. The distinct immune system of the liver and the resulting alterations in the immune-mediated microenvironment of tumor cells serve as key factors in the advancement of hepatocellular carcinoma [28–30]. Because of the absence of appropriate systemic remedies and the distinct immune-mediated conditions in the liver, targeted therapy and immunotherapy has been extensively studied in unresectable hepatocellular carcinoma through clinical trials. Unfortunately, not all hepatocellular carcinoma patients respond to immunotherapy. Heterogeneity in tumor antigens within the same tumor, between tumors in the same patient, and among different patients poses challenges for immune therapy. Tumor heterogeneity has led to the exploration of various immune checkpoint inhibitors (ICIs) targets and adoptive cell therapies. Nevertheless, there is no foolproof way to predict which patients would react. In this study, we developed two predictive models for atezolizumab plus bevacizumab in unresectable hepatocellular carcinoma based on ultrasound imaging-based radiomics. Both models demonstrated excellent predictive potential for treatment response and survival prediction, with the post-treatment model showing greater significance.

With the emergence of new targeted and immune therapies, methods for predicting treatment efficacy are rapidly evolving. By analyzing gene expression data from tumor tissue or blood, specific genes or genomic features associated with response to particular therapeutic drugs can be identified, and the expression levels of specific target genes can be measured to predict the efficacy of certain targeted drugs [31,32]. Changes in specific protein biomarkers can also be used to predict the efficacy of certain targeted treatments [33]. Currently, companion diagnostics (CDx) for targeted and immune therapies in clinical practice for hepatocellular carcinoma primarily involve testing for PD-1/PD-L1 expression. PD-1 is mainly expressed on the surface of immune cells, while PD-L1 is highly expressed on most tumor cells [34,35], and detection methods include immunohistochemistry (IHC). Combining decreased tumor stiffness and increased AFP levels may provide potential valuable biomarkers for Glypican-3 (GPC3)-targeted therapy and immunotherapy [36]. Imaging findings from hepatocellular carcinoma patients’ radiological examinations (such as CT scans, MRI, etc.) can be used to construct models for predicting the efficacy of targeted therapy. These models utilize machine learning algorithms and image analysis techniques to extract features relevant to treatment response and make predictions [37–39].

Previous studies predicting treatment efficacy mostly focused on pre-treatment models. In this study, for the first time, we used ultrasound imaging-based radiomics to predict treatment response both before and after treatment. Results from previous studies demonstrated that the first confirmed responses to atezolizumab plus bevacizumab are mostly observed at the time of three months after initiating treatment. Therefore, the pre- and post-treatment (after three months) ultrasound images were employed. The results showed that the AUCs of the pre-treatment UR model and post-treatment UR model were 0.790 (95% CI: 0.701–0.878) and 0.855 (95% CI: 0.780–0.931) respectively (Fig 4), indicating good predictive value for both models, with the post-treatment model showing greater significance. Additionally, since the efficacy of targeted therapy and immunotherapy in unresectable hepatocellular carcinoma is closely related to patient survival, we made predictions of overall survival (OS) and constructed a nomogram. The C-index for the pre-treatment and post-treatment models was 0.720 and 0.734, respectively, calculated without weighting, with significant differences between the two. Consistent with the prediction of treatment efficacy, the post-treatment radiomics had a greater predictive value for survival, further confirming the significant extension of survival in patients who responded well to targeted therapy and immunotherapy.

This study confirmed the high value of radiomics in evaluating the efficacy of targeted therapy and immunotherapy in unresectable hepatocellular carcinoma. Numerous previous studies have demonstrated that texture analysis in radiomics can quantitatively analyze the texture features of images to assess the impact of treatment on tumors. This method can capture subtle tissue structural changes, thereby providing a more sensitive and comprehensive evaluation of treatment effects [40–42]. Changes in post-treatment ultrasound radiomics can be attributed to variations in tumor stiffness caused by targeted therapy and immunotherapy, as well as cellular damage during the systemic treatment process, leading to tumor necrosis [43,44]. Research has shown that parameters based on radiomic analysis of skeletal muscle and adipose tissue can predict a year longevity rate for people suffering from unresectable hepatocellular carcinoma [45]. In patients treated with SIRT and sorafenib, radiomic-based parameters have higher prognostic value. Therefore, the decrease in tumor cell density, which may result in tumor softening, can be detected through changes in imaging. In other words, radiomics captures the reduction in tumor hardness, indicating that the tumor becomes softer due to necrotic development following targeted therapy and immunotherapy, suggesting the effectiveness of the immunotherapeutic regimen. Moreover, this information might be evident as early as one week after treatment, significantly earlier than the conventional one-month assessment post-treatment. This early prediction of non-response to targeted therapy and immunotherapy can benefit patients with advanced-stage disease as it provides an opportunity to explore alternative comprehensive treatment approaches that may be effective instead of continuing with the initial ineffective regimen observed during follow-up examinations conducted approximately two months later. Therefore, we recommend that patients with unresectable hepatocellular carcinoma who are not eligible for surgery undergo ultrasound radiomics evaluation of changes one week after starting targeted therapy and immunotherapy, as these changes may serve as biomarkers for predicting immunotherapeutic response, aiding in the early assessment of immunotherapy responsiveness. Additionally, targeted therapy and immunotherapy can also cause liver injury, which may affect liver stiffness and consequently impact the measurement of tumor hardness [46].

### Limitation

The study has a number of drawbacks. Firstly, it is a single-center retroactive study. Secondly, there was no pathological correlation performed, as obtaining tissue samples from patients with unresectable hepatocellular carcinoma undergoing targeted therapy and immunotherapy is not feasible in routine clinical practice. Thirdly, there is a lack of comparison at multiple time points after treatment, therefore the suitability of assessing efficacy one week post-surgery needs further validation. Lastly, there is heterogeneity in the targeted therapy and immunotherapy regimens used.

## Conclusion

We have developed and validated two models for predicting the efficacy of targeted therapy and immunotherapy. The ultrasound image-based features obtained one week post-surgery showed better predictive performance and can be used to guide clinical treatment decisions, allowing patients who are unlikely to benefit from targeted therapy and immunotherapy to explore alternative treatment options and maximize their clinical outcomes.

## Supporting information

S1 File Raw data. (RAR)
